# Intentional spatial coding of responses under multiple action effect situations

**DOI:** 10.3389/fpsyg.2023.1164500

**Published:** 2023-08-24

**Authors:** Loïc P. Heurley, Laurent P. Ferrier, Alexandre Coutté, Guillaume Thébault

**Affiliations:** ^1^Laboratoire sur les Interactions Cognition, Action, Émotion (LICAE)—Université Paris Nanterre, Nanterre, France; ^2^Laboratoire Interdisciplinaire de Recherche en Didactique, Éducation, Formation (LIRDEF 3749), Atelier Projet NEXUS, Université Montpellier 3, Montpellier, France; ^3^Epsylon (EA 4556), Université Montpellier 3, Montpellier, France; ^4^Centre Hospitalier Paul Coste Floret, Lamalou-les-Bains, France

**Keywords:** action representation, body representation, action effects, spatial coding, intention

## Introduction

A major question in cognitive sciences concerns the nature of neurocognitive processes underlying the representation of the body in action. Since the first proponents of the ideomotor view (for a historical review, see Stock and Stock, 2004), special attention has been devoted to the perceptual consequences of actions or action effects. A recent development of this view, the theory of event coding (Hommel et al., 2001), assumed that the spatial features of action effects, which are perceptual in nature, would be an elementary component of action representations (for review and supporting evidence, see Hommel, 2015, 2019). A fruitful way to investigate these kinds of components lies in the stimulus–response compatibility effect as the Simon effect (Simon and Rudell, 1967). In these tasks, participants exhibit faster RTs when the location of a target stimulus and the response match (e.g., right stimulus/right response) rather than mismatch (e.g., left stimulus/right response) even when the task does not require us to discriminate the spatial locations of stimuli and responses (for a review, see Proctor and Vu, 2006). This effect is explained by the compatibility of spatial (horizontal) codes dedicated to stimuli and responses (Wallace, 1971; Nicoletti and Umiltà, 1984). According to Hommel (2011), the coding of responses along the horizontal axis occurs because the right and left responses usually induce action effects on the right and left sides, respectively. Among the possible action effects involved are the visual movements of the index fingers pressing the switches, the tactile feedback of the switches under the pulp of the fingers, the proprioceptive feedback of the hands/fingers moving, and the auditory feedback of the switches that are pressed and depressed. Therefore, the Simon paradigm allows us to investigate how people code their responses based on their action effects, providing a window into their more general ability to represent actions.

As recently advocated by Pfister (2019), one question becomes of primary importance in this general account: How can people cognitively deal with situations in which a specific action induces various spatially non-congruent action effects at the same time? It is widely acknowledged that each action produces various perceptual effects simultaneously (with visual, tactile, and proprioceptive effects being the most ubiquitous, Tsakiris, 2010). As a result, people are consistently immersed in situations requiring them to handle multiple effects and must consider the relationship between their actions and their effects. Addressing this question is thus a crucial requirement to better understand the dynamic of action representations in complex environments. Furthermore, it has important implications for questions related to (i) the construction of the body schema and the emergence of a sense of body ownership (Ehrsson, 2020), (ii) the development of a sense of agency (Haggard and Clark, 2003), and (iii) the effective control of actions (e.g., Fleury et al., 2019). Over the past 40 years, this question has been sporadically addressed. This article summarizes studies on this subject and outline two major ideas: first, we argue that the spatial coding of responses, beyond the representation of the body in action, primarily depends on the intention of the agent. The involvement of intention-based processes particularly resonates with recent developments, suggesting that integrating multiple effects necessitates a complex interplay between bottom–up and top-down processes (Blanke et al., 2015). Second, these studies allow us to sketch a general method that is appropriate for investigating the nature of these intentional-based processes. This method holds great significance as it could inspire the development of innovative methodologies that bridge the fields of body schema and action representation: a necessary step forward for both domains.

## The manipulation of artificial action effects

A number of studies have combined a Simon task and responses inducing spatially non-congruent natural and artificial action effects. In Hommel (1993), participants were instructed to press the right/left switch that flashed a red light located, respectively, on the left and right sides (i.e., opposite sides). Pressing the right key induced natural action effects (e.g., visual effects of the hands moving or tactile feedback of the index pressing the switches), all on the right side, while the artificial visual effect,^1^ the red light, appeared on the opposite (left) side. Hommel (1993) also manipulated the way participants conceived the task: they had to either press the switches or flash the light according to the tone of auditory stimuli (i.e., auditory Simon task). Data showed that when the participants intended to press the switch, the Simon effect was driven by the compatibility between the locations of stimuli and switches. Conversely, when the participants intended to flash the light, the Simon effect was driven by the compatibility between the locations of stimuli and lights. These results suggest that the coding of responses is malleable as it depends on various action effects as a function of the goal of the participants. Hommel (1996) used a complementary protocol in which the right/left response induced an auditory tone located on the same side for one group (i.e., spatially congruent group) or on the opposite side for the other group (i.e., spatially non-congruent group). The task was similar for both groups. They had to press the relevant switch according to the color of the visual stimuli. Hommel (1996) observed that, for both groups, the Simon effect was driven by the compatibility between the location of stimuli and switches. However, in the spatially non-congruent group, the magnitude of the Simon effect was smaller compared to the spatially congruent group. This result is in line with the study by Hommel (1993). When the task highlighted the relevance of the location of the switches, this component predominated. However, in the spatially non-congruent condition, the decrease in the magnitude of the Simon effect could be interpreted as a diminishing the lateral coding of responses because of the integration of the (opposite) locations of the auditory effects of responses (for replication and extension, see Grosjean and Mordkoff, 2002). In accordance, it seems possible that various action effects could be integrated at the same time. However, this last conclusion should be nuanced, considering the studies detailed below.

## A steering wheel as a response device

Guiard (1983) developed another kind of method that involves using a steering wheel to respond to an auditory Simon task. Participants of the first group had to put their hands on the top of the steering wheel, while the other group placed their hands on the bottom part of the apparatus. Hence, in this last condition, when the steering wheel was moved toward the right/left, the hands moved in the opposite direction. Accordingly, the action effects associated with the steering wheel and with the hands were spatially non-congruent. Guiard (1983) found a non-significant Simon effect in this group. Interestingly, he went further and observed some heterogeneity in his sample with five participants who exhibited a Simon effect driven by the stimuli/hand compatibility and three participants who exhibited a (reverse) effect driven by the stimuli/wheel compatibility. It is noteworthy that Guiard (1983) used instructions that did not highlight an effect in particular. Indeed, he solely mimicked how the participants had to respond to the apparatus. As a result, it is possible to argue that, in this experiment, the intention of the participants was not controlled. Some participants might intend to move the steering wheel while others might intend to move their hands. They thus integrated, respectively, the direction of the steering wheel or the direction of the hands to code their responses. This opposite coding may have induced, in turn, opposite Simon effects that canceled out each other when they were averaged. Wang et al. (2003) replicated these findings and also found a lack of Simon effects when the hands were put on the bottom part of the steering wheel. Among their 32 participants, 18 exhibited a Simon effect driven by the stimuli/hand compatibility, while 14 exhibited a (reverse) effect driven by the stimuli/wheel compatibility. To overcome the canceling of the effect when data were averaged, Wang et al. (2003) calculated the magnitude of the Simon effect by averaging the absolute values of individual effects and found a 42-ms effect size. A size quite similar to the one reported in the group putting their hand on the top part of the steering wheel. This last result supports that two kinds of Simon effects may indeed coexist in their sample. Wang et al. (2007) consistently observed a Simon effect driven by the stimuli/hand compatibility when instructions emphasized hand movements rather than steering wheel movements, supporting the conclusion that the spatial coding of responses depends on the action effects of hands rather than of the steering wheel.

## The reverse of natural visual feedback

The last method consists of rendering some natural action effects of the response's hands spatially non-congruent, as indicated in the study of Sutter and Ladwig (2012). Their participants saw their hands through a screen reversing online the visual feedback of their hands along the horizontal axis.^2^ Therefore, when they used their right hand to respond, they saw their hand moving on the left side as if it was their left hand. In this setup, the natural visual effects of the participants' hands were spatially non-congruent with the tactile and proprioceptive effects. The results showed a Simon effect mainly driven by the compatibility between the location of stimuli and the location of tactile and proprioceptive effects rather than the visual ones. Additionally, Sutter and Ladwig (2012) found that this Simon effect increased over time (i.e., along five blocs), possibly suggesting that initially, a competition between various effects occurred to code the responses. Then, progressively, considering the constraints of the task, the participants preferentially based their coding on tactile/proprioceptive effects. In addition, the magnitude of the Simon effect in this condition was smaller than the one in the spatially congruent condition (i.e., non-reverse condition). We can accordingly speculate that the experimental setup as well as the task used possibly allowed participants to use different strategies to resolve the task that, in turn, modulated the action effects used to code the responses. Nevertheless, as these authors did not precisely detail the task, they neither reported individual Simon effects nor an analysis of the magnitude of the Simon effect based on absolute values, and it was difficult to confirm such an interpretation.

## Toward a predictive-based account and individual-based analyses

These experiments collectively indicate the significance of an agent's intention in constructing action representations. This overarching concept has been previously suggested by various studies emphasizing how action representations rely on the goals of the actions (e.g., Riggio et al., 1986; Buhlmann et al., 2007; Cattaneo et al., 2009; Rochat et al., 2010; Memelink and Hommel, 2013; Osiurak and Badets, 2014). However, the studies discussed in this article specifically investigate how intention influences the selection of action effects, representing a distinct way in which intention can drive action representation. Thus, the same action can result in different spatial codings if the agent conceives its goal differently. This conclusion aligns with recent predictive-based theories of multimodal integration, such as predictive coding (Clark, 2013) and the free-energy principle (Friston, 2010). These theories argue that solely considering bottom–up processes is insufficient to fully explain the range of phenomena related to multimodal integration, including body-ownership illusions (Blanke et al., 2015; Ehrsson, 2020). Instead, there would exist a complex interplay between top-down processes, involving the generation of predictions about incoming sensory inputs, and bottom-up processes dedicated to processing the actual sensory inputs. The neurocognitive system strives to minimize the disparities between predictions and inputs (Pezzulo et al., 2021, 2022). In line with this, our main proposition is that the intention-based processes involved in the spatial coding of responses can be seen as top-down processes that modulate the generated predictions. Accordingly, the Simon effect and the protocols described in this opinion study can be viewed as a means to gain insights into the processes underlying the generation of predictions and their modulation through top-down processes.

Despite the theoretical significance of this proposal, the very nature of intentional-based processes remains largely unexplored. One possible explanation for this gap lies in the lack of a suitable method to properly capture the intentional-based processes. In particular, the reviewed literature on spatial coding reveals a significant challenge: the considerable inter-individual variability in intentional-based processes even when experimental protocols remain similar. It is worth noting that task instructions appear to be a powerful means of modulating agents' intentions (e.g., Hommel, 1993; Wang et al., 2007). However, in some cases, task instructions may not completely prevent participants from adopting different intentions (e.g., Guiard, 1983; Wang et al., 2003). Instead of seeking to eliminate this inherent inter-individual variability, which is fundamental to intentional processes, future studies should develop alternative methods to address and account for this variability. We argue that special attention should be given to two aspects: (i) the magnitude of compatibility effects both in relative and absolute values and (ii) individual effects. Averaging compatibility effects across an entire sample can mask the use of different strategies. For example, a reduction in the magnitude of the Simon effect (e.g., Hommel, 1996; Sutter and Ladwig, 2012) or a null or non-significant effect (e.g., Guiard, 1983; Wang et al., 2003) could actually be attributed to differing intentions among participants. This proposal aligns with a recent methodological suggestion by Speelman and McGann (2020), Moore et al. (2023). These authors advocate for the importance of pervasiveness analysis, which involves examining the number of individuals who exhibit the effect under investigation, going beyond mere data averaging in the studied sample. We argue that this method should be consistently employed when investigating intentional processes.

## Author contributions

LH wrote the first draft. LF, AC, and GT participated to the correction and improvement of the manuscript. All authors contributed to the article and approved the submitted version.

## Footnotes

^1^This effect is qualified as “artificial” because it is induced by the experimenter rather than to be a natural outcome of body movements in interaction with the physical world.

^2^Sutter and Ladwig (2012) also transformed the visual feedback along the y-axis. We nevertheless did not develop this condition further because, in this case, action effects were not spatially non-congruent along the horizontal axis.

## References

[B1] Blanke O. Slater M. Serino A. (2015). Behavioral, neural, and computational principles of bodily self-consciousness. Neuron 88, 145–166. 10.1016/j.neuron.09029 26447578

[B2] Buhlmann I. Umiltà C. Wascher E. (2007). Response coding and visuomotor transformation in the Simon task: the role of action goals. J. Exp. Psychol. Human Percep. Perform. 33, 1269–1282. 10.1037/0096-33,6.1269 18085942

[B3] Cattaneo L. Caruana F. Jezzini A. Rizzolatti G. (2009). Representation of goal and movements without overt motor behavior in the human motor cortex: a transcranial magnetic stimulation study. J. Neurosci. 29, 11134–11138. 10.1523/JNEUROSCI.2605-09.2009 19741119 PMC6665924

[B4] Clark A. (2013). Whatever next? Predictive brains, situated agents, and the future of cognitive science. Behav. Brain Sci. 36, 181–204. 10.1017/S0140525X12000477 23663408

[B5] Ehrsson H. H. (2020). “Multisensory processes in body ownership,” in Multisensory Perception: From Laboratory to Clinic, eds K. Sathian and V. S. Ramachandran (Cambridge, MA: Academic Press), 179–200. 10.1016/b978-0-12-812492-5.00008-5

[B6] Fleury L. Prablanc C. Priot A. E. (2019). Do prism and other adaptation paradigms really measure the same processes? Cortex 119, 480–496. 10.1016/j.cortex.07012 31525564

[B7] Friston K. (2010). The free-energy principle: a unified brain theory? Nat. Rev. Neurosci. 11, 127–138. 10.1038/nrn2787 20068583

[B8] Grosjean M. Mordkoff J. T. (2002). Post-response stimulation and the Simon effect: further evidence of action-effect integration. Visual Cogn. 9, 528–539. 10.1080/13506280143000566

[B9] Guiard Y. (1983). The lateral coding of rotations: a study of the Simon effect with wheel-rotation responses. J. Motor Behav. 15, 331–342. 10.1080/002198310735303 15151865

[B10] Haggard P. Clark S. (2003). Intentional action: conscious experience and neural prediction. Conscious. Cogn. 12, 695–707. 10.1016/S1053-8100(03)00052-7 14656511

[B11] Hommel B. (1993). Inverting the Simon effect by intention: determinants of direction and extent of effects of irrelevant spatial information. Psychol. Res. 55, 270–279. 10.1007/BF00419687

[B12] Hommel B. (1996). The cognitive representation of action: automatic integration of perceived action effects. Psychol. Res. 59, 176–186. 10.1007/BF00425832 8923816

[B13] Hommel B. (2011). The Simon effect as tool and heuristic. Acta Psychol. 136, 189–202. 10.1016/j.actpsy.2010.04.011 20507830

[B14] Hommel B. (2015). The theory of event coding (TEC) as embodied-cognition framework. Front. Psychol. 6, 1318. 10.3389/fpsyg.2015.01318 26388819 PMC4554939

[B15] Hommel B. (2019). Theory of Event Coding (TEC) V2, 0. Representing and controlling perception and action. Attent. Percep. Psychophys. 81, 2139–2154. 10.3758/s13414-019-01779-4 31168699 PMC6848055

[B16] Hommel B. Müsseler J. Aschersleben G. Prinz W. (2001). The theory of event coding (TEC): a framework for perception and action planning. Behav. Brain Sci. 24, 849–878. 10.1017/S0140525X01000103 12239891

[B17] Memelink J. Hommel B. (2013). Intentional weighting: a basic principle in cognitive control. Psychol. Res. 77, 249–259. 10.1007/s00426-012-0435-y 22526717 PMC3627030

[B18] Moore S. Speelman C. P. McGann M. (2023). Pervasiveness of effects in sample-based experimental psychology: a re-examination of replication data from nine famous psychology experiments. New Ideas Psychol. 68, 100978. 10.1016/j.newideapsych.2022.100978

[B19] Nicoletti R. Umiltà C. (1984). Right-left prevalence in spatial compatibility. Percept. Psychophys. 35, 333–343. 10.3758/BF03206337 6739268

[B20] Osiurak F. Badets A. (2014). Pliers, not fingers: tool-action effect in a motor intention paradigm. Cognition 130, 66–73. 10.1016/j.cognition.09005 24184395

[B21] Pezzulo G. Parr T. Friston K. (2022). The evolution of brain architectures for predictive coding and active inference. Philosophical transactions of the royal society of London. Series B Biol. Sci. 377, 20200531. 10.1098/rstb.2020.0531 34957844 PMC8710884

[B22] Pezzulo G. Zorzi M. Corbetta M. (2021). The secret life of predictive brains: what's spontaneous activity for? Trends Cogn. Sci. 25, 730–743. 10.1016/j.tics.05007 34144895 PMC8363551

[B23] Pfister R. (2019). Effect-based action control with body-related effects: implications for empirical approaches to ideomotor action control. Psychol. Rev. 126, 153–161. 10.1037/rev0000140 30604990

[B24] Proctor R. W. Vu K. P. L. (2006). Stimulus-Response Compatibility Principles: Data, Theory, and Application. Boca Raton, FL: CRC press.

[B25] Riggio L. Gonzaga Gawryszewski d. e. Umilta L. (1986). What is crossed in crossed-hand effects? Acta Psychol. 62, 89–100. 10.1016/0001-6918(86)90006-5

[B26] Rochat M. J. Caruana F. Jezzini A. Escola L. Intskirveli I. Grammont F. (2010). Responses of mirror neurons in area F5 to hand and tool grasping observation. Exp. Brain Res. 204, 605–616. 10.1007/s00221-010-2329-9 20577726 PMC2903687

[B27] Simon J. R. Rudell A. P. (1967). Auditory SR compatibility: the effect of an irrelevant cue on information processing. J. Appl. Psychol. 51, 300–304. 10.1037/h0020586 6045637

[B28] Speelman C. P. McGann M. (2020). Statements about the pervasiveness of behavior require data about the pervasiveness of behavior. Front. Psychol. 11, 594675. 10.3389/fpsyg.2020.594675 33329258 PMC7711086

[B29] Stock A. Stock C. (2004). A short history of ideo-motor action. Psychol. Res. 68, 176–188. 10.1007/s00426-003-0154-5 14685855

[B30] Sutter C. Ladwig S. (2012). Mirrored visual feedback limits distal effect anticipation. Exp. Brain Res. 218, 247–258. 10.1007/s00221-012-3018-7 22331170

[B31] Tsakiris M. (2010). My body in the brain: a neurocognitive model of body-ownership. Neuropsychologia 48, 703–12. 10.1016/j.neuropsychologia.09034 19819247

[B32] Wallace R. J. (1971). SR compatibility and the idea of a response code. J. Exp. Psychol. 88, 354–360. 10.1037/h0030892 5090926

[B33] Wang D. Y. D. Proctor R. W. Pick D. F. (2003). The Simon effect with wheel-rotation responses. J. Motor Behav. 35, 261–273. 10.1080/00222890309602139 12873841

[B34] Wang D. Y. D. Proctor R. W. Pick D. F. (2007). Coding controlled and triggered cursor movements as action effects: Influences on the auditory Simon effect for wheel-rotation responses. J. Exp. Psychol. Human Percep. Perform. 33, 657–669. 10.1037/0096-33,3.657 17563228

